# Managing fruit rot diseases of *Vaccinium corymbosum*

**DOI:** 10.3389/fpls.2024.1428769

**Published:** 2024-08-06

**Authors:** Kerri A. Neugebauer, Chakradhar Mattupalli, Mengjun Hu, Jonathan E. Oliver, Joshua VanderWeide, Yuzhen Lu, Kevin Sullivan, Virginia O. Stockwell, Peter Oudemans, Timothy D. Miles

**Affiliations:** ^1^ Department of Plant, Soil, and Microbial Sciences, Michigan State University, East Lansing, MI, United States; ^2^ Department of Plant Pathology, Washington State University, Northwestern Washington Research and Extension Center, Mount Vernon, WA, United States; ^3^ Department of Plant Science and Landscape Architecture, University of Maryland, College Park, MD, United States; ^4^ Department of Plant Pathology, University of Georgia, Tifton, GA, United States; ^5^ Department of Horticulture, Michigan State University, East Lansing, MI, United States; ^6^ Department of Biosystems and Agriculture Engineering, Michigan State University, East Lansing, MI, United States; ^7^ Office of Research Analytics, New Jersey Agricultural Experiment Station, Rutgers, Rutgers University, New Brunswick, NJ, United States; ^8^ Horticultural Crops Disease and Pest Management Research Unit, United States Department of Agriculture, Agricultural Research Service, Corvallis, OR, United States; ^9^ Department of Plant Biology, Philip E. (P.E) Marucci Center for Blueberry and Cranberry Research and Extension, Rutgers University, Chatsworth, NJ, United States

**Keywords:** anthracnose, Botrytis fruit rot, *Colletotrichum* spp., *Botrytis cinerea*, highbush blueberry

## Abstract

Blueberry is an important perennial fruit crop with expanding consumption and production worldwide. Consumer demand for blueberries has grown due to the desirable flavor and numerous health benefits, and fresh market production in the U.S. has risen in turn. U.S. imports have also increased to satisfy year-round consumer demand for fresh blueberries. Pre- and post-harvest fruit diseases such as anthracnose (caused by *Colletotrichum* spp.) and botrytis fruit rot (caused by *Botrytis* spp.) have a significant impact on fruit quality and consumer acceptance. These are also among the most difficult diseases to control in the blueberry cropping system. These latent pathogens can cause significant losses both in the field, and especially during transport and marketplace storage. Although both diseases result in rotted fruit, the biology and infection strategies of the causal pathogens are very different, and the management strategies differ. Innovations for management, such as improved molecular detection assays for fungicide resistance, postharvest imaging, breeding resistant cultivars, and biopesticides have been developed for improved fruit quality. Development and integration of new strategies is critical for the long-term success of the blueberry industry.

## 1 Introduction

Blueberry is a high-value perennial crop native to North America and grown throughout the world. There are approximately 450 species of plants referred to as “blueberry”, but those most grown in the U.S. are highbush blueberry, lowbush blueberry, half-high, and rabbiteye (Fulcher et al., 2015). Highbush blueberries are separated into the northern (*Vaccinium corymbosum* L.) and southern highbush blueberry. Southern highbush blueberries are interspecific hybrids of northern highbush and *Vaccinium* species found in the southeast U.S. including evergreen (*V. darrowii*), rabbiteye (*V. virgatum*), or lowbush (*V. angustifolium* Aiton and *V. myrtilloides* Michx.) (Fulcher et al., 2015; Retamales and Hancock, 2018). Highbush and rabbiteye blueberries are mostly cultivated as traditional field grown perennial crops, while lowbush blueberries are managed in native stands (Adaskaveg et al., 2022). Among all commercially produced blueberry species, northern highbush blueberry comprises the majority of U.S. production, with over 75% of the cultivated area (Rodriguez-Saona et al., 2019).

The volume of highbush blueberry production has continuously increased and expanded since the early 2000’s (Yeh et al., 2023). In order to meet the now year-round consumer demand, both the U.S. blueberry production as well as imports have increased (Kramer, 2020). Within the 20-year period from 2002 to 2022, U.S. highbush blueberry acreage increased by 134%, from 21,044 to 49,285 hectares USDA, National Agricultural Statistics Service (2023). Not surprisingly, the total U.S. production in 2000-2002, increased by 283% from 41 million kilograms of fresh blueberries to 156 million kg in 2018-2020 (Yeh et al., 2023). During the same periods, a 408% increase in importation to the U.S. occurred and by 2018-2020, a total of 182 million kg of blueberries were imported (Yeh et al., 2023). In 2020, about 97% of these imports came from Peru, Chile, Mexico, and Canada (Weber et al., 2023; Yeh et al., 2023). Although imported fruit can insure 12-month availability, fruit received in September and October from Peru directly increases competition for producers in Michigan, Washington, and Oregon (Weber et al., 2023). Similarly, imports from Chile and Mexico have increased competition for California, Florida, and Georgia as fruit can be imported through the winter months ending in early May and June (Yeh et al., 2023).

**Figure 1:**
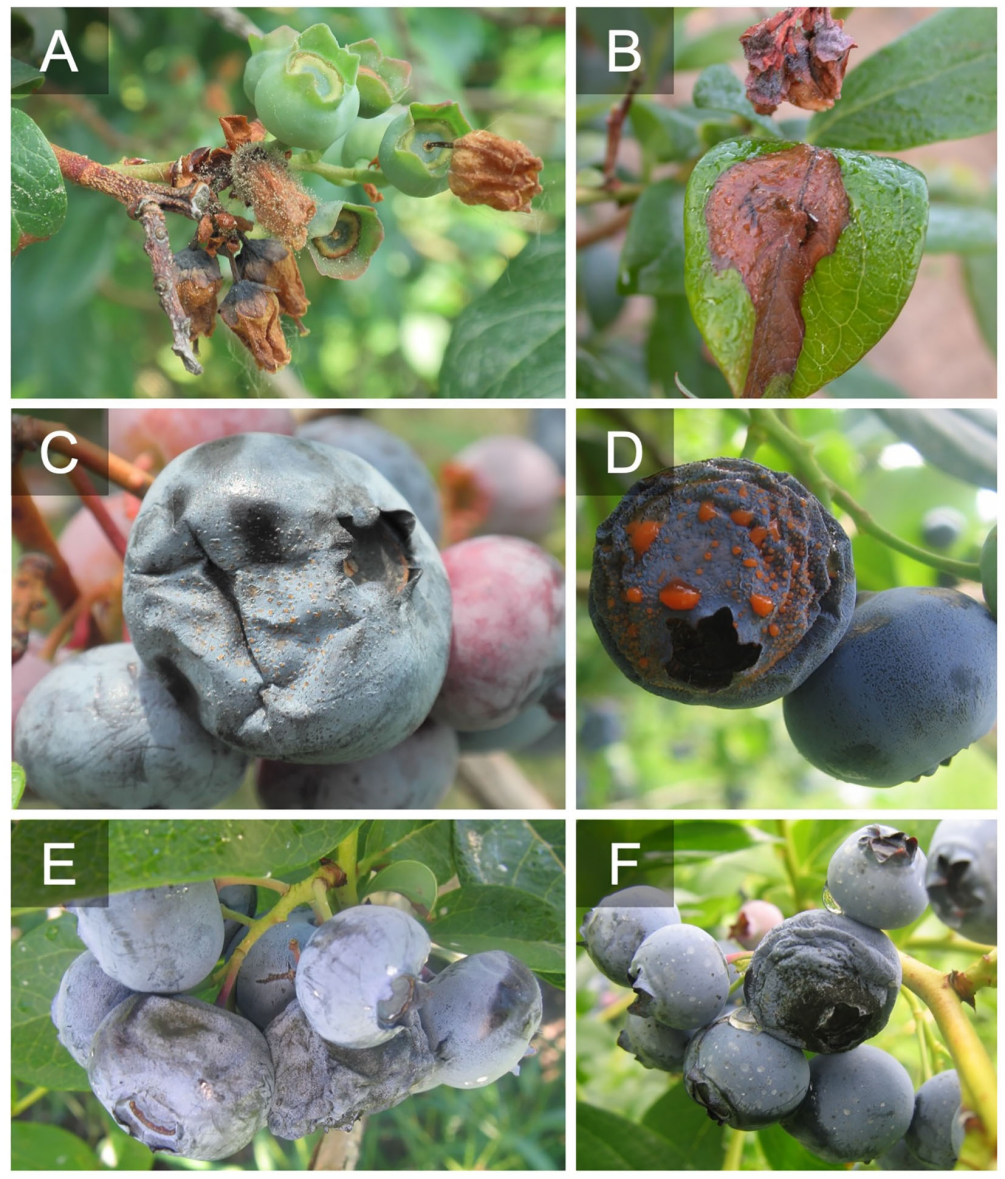
Botrytis blossom blight and shoot blight caused by *Botrytis cinerea* **(A, B)**. Acervuli and sporulation of *Colletotrichum fioriniae* on ripe blueberries **(C, D)**. Alternaria fruit rot caused by *Alternaria* spp. on ripe fruit on the fruit surface and around the calyx cup **(E, F)**.

**Figure 2:**
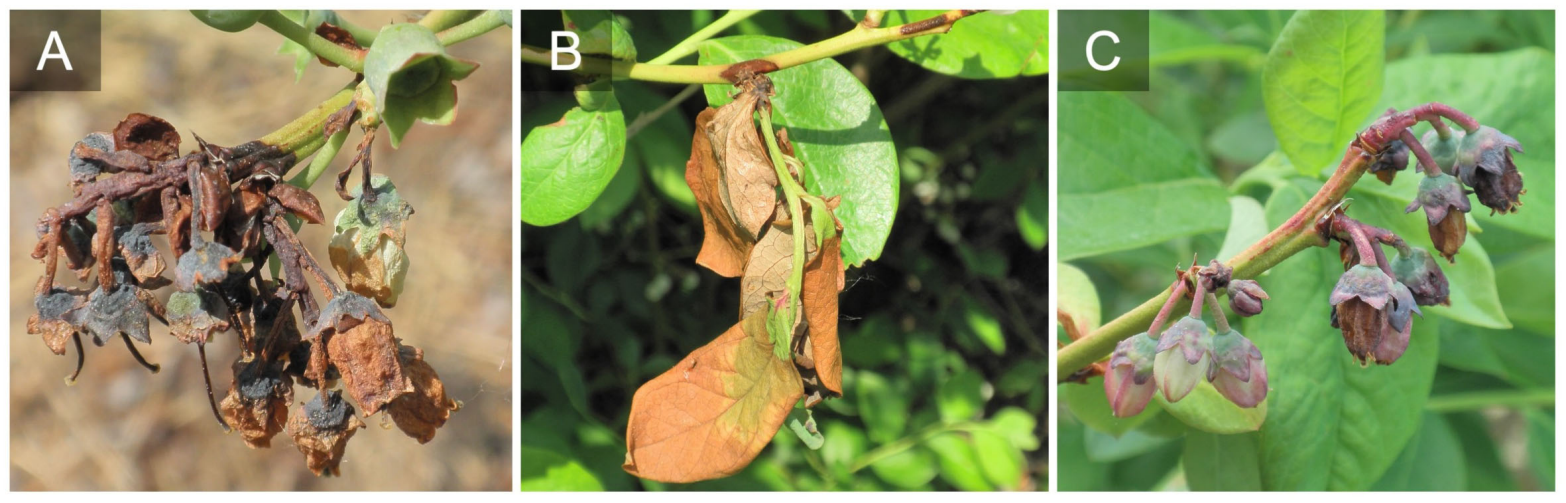
Look alike symptoms probably caused by other pathogens or factors. Blossom and foliar blight caused by some type of stem dieback either freeze damage or a stem pathogen like *Diaporthe* spp., note the shoot necrosis **(A, B)**. Aborted blossoms that present as a blight. This could be a pathogen like *Botrytis cinerea* that isn’t sporulating or another factor such as freeze damage **(C)**.

**Figure 3:**
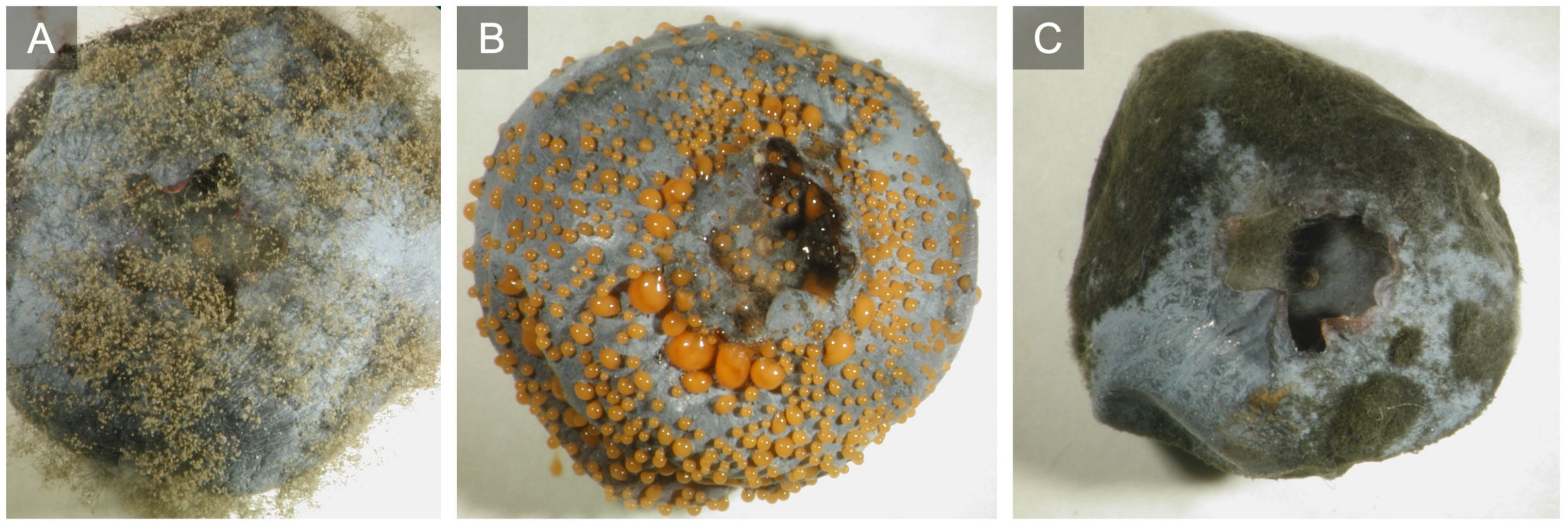
Postharvest signs of botrytis fruit rot **(A)**, anthracnose fruit rot **(B)**, and alternaria fruit rot **(C)** after incubation at high relative humidity.

With this increased competition, blueberry growers in the U.S. are striving to increase yield, improve fruit quality, and extend postharvest shelf-life to remain competitive. However, blueberries are susceptible to a variety of fruit rots that can cause yield loss and reduce fruit quality postharvest. Botrytis fruit rot (caused by *Botrytis* spp.) and anthracnose fruit rot (caused by *Colletotrichum* spp.) as well as alternaria fruit rot (caused by *Alternaria* spp.) are the most economically important diseases in highbush blueberry in North America (**Figure 1**). These diseases are typically managed with multiple prophylactic fungicide applications throughout the growing season from pre-bloom through harvest (Wise et al., 2020). If left unmanaged, significant fruit rot can occur pre- and post-harvest. Disease diagnosis is critical since management tactics differ depending on the disease and can be challenging in the field without observing signs of the pathogen i.e., sporulation (**Figure 2**). Yield losses due to anthracnose fruit rot have been reported as high as 10-20% in well-managed fields, while postharvest losses during storage can reach up to 100% (Milholland and Schilder, 2017) (**Figure 3**). Botrytis fruit rot and its associated blossom blight is more episodic with a potential to cause yield losses as high as 30-40% (Bristow et al., 2017). However, the occurrence of fungicide resistance is increasing in these pathogens, causing control failures, and making disease management challenging. Additionally, with the import market growing and time in storage increasing, controlling postharvest pathogens and maintaining fruit quality are becoming difficult (Castro et al., 2023). This review will detail the biology of these major fruit rot pathogens, molecular detection tools to identify the pathogens, and management strategies.

## 2 Botrytis fruit rot

### 2.1 Causal organism

*Botrytis cinerea* Pers., the causal agent of gray mold, blossom blight, and botrytis fruit rot, is a necrotrophic fungus that is a major pathogen in many economically important crops worldwide, including grapes, strawberries, apples, and blueberries (Saito et al., 2016; Naegele et al., 2022). This fungus was identified as the second most important fungal plant pathogen based on its broad host range and scientific and economic importance (Dean et al., 2012). *Botrytis cinerea* is considered a generalist as it has the widest host range of all *Botrytis* species and can infect more than 1,400 plant species worldwide (Staats et al., 2005; Saito et al., 2017; Garfinkel et al., 2019). It is estimated that *B. cinerea* causes over $10 billion in annual economic losses worldwide (Hua et al., 2018).

Within *B. cinerea*, two groups (Group I and Group II) were proposed and differentiated by the presence or absence of two transposable elements (TEs), *Boty* (Diolez et al., 1995) and *Flipper* (Fournier et al., 2003; Fournier et al., 2005; Levis et al., 1997). It was later shown using multi-locus sequencing analysis that these groups are two distinct species (Walker et al., 2011) designated as *B. pseudocinerea* (former Group I) and *B. cinerea sensu stricto* (former Group II). Although the species are morphologically indistinguishable, there are differences in fungicide sensitivity (Walker et al., 2011; DeLong et al., 2020). *B. pseudocinerea* has a natural resistance to fenhexamid, a fungicide commonly used to control botrytis fruit rot (Fournier et al., 2003) and exhibits hypersensitivity to fenpropidin (DeLong et al., 2020). A new species within the *B. cinerea* species complex, *B. californica*, was described using multi-locus sequencing and was found to cause disease on blueberry in California (Saito et al., 2017). *B. californica* can be distinguished visually as it has longer conidiophores than *B. cinerea* and *B. pseudocinerea* (Saito et al., 2017). Although *B. pseudocinerea* and *B. californica* both can cause disease on blueberries, the predominant disease-causing species is *B. cinerea* (Saito et al., 2014; Adaskaveg et al., 2022). Interspecific crosses have been observed in *Botrytis* spp. and gene flow between species may happen, however only crosses between individuals of the same species will produce apothecia (Nielsen and Yohalam, 2001; Walker et al., 2011; Saito et al., 2017).

### 2.2 Lifecycle

**Figure 4:**
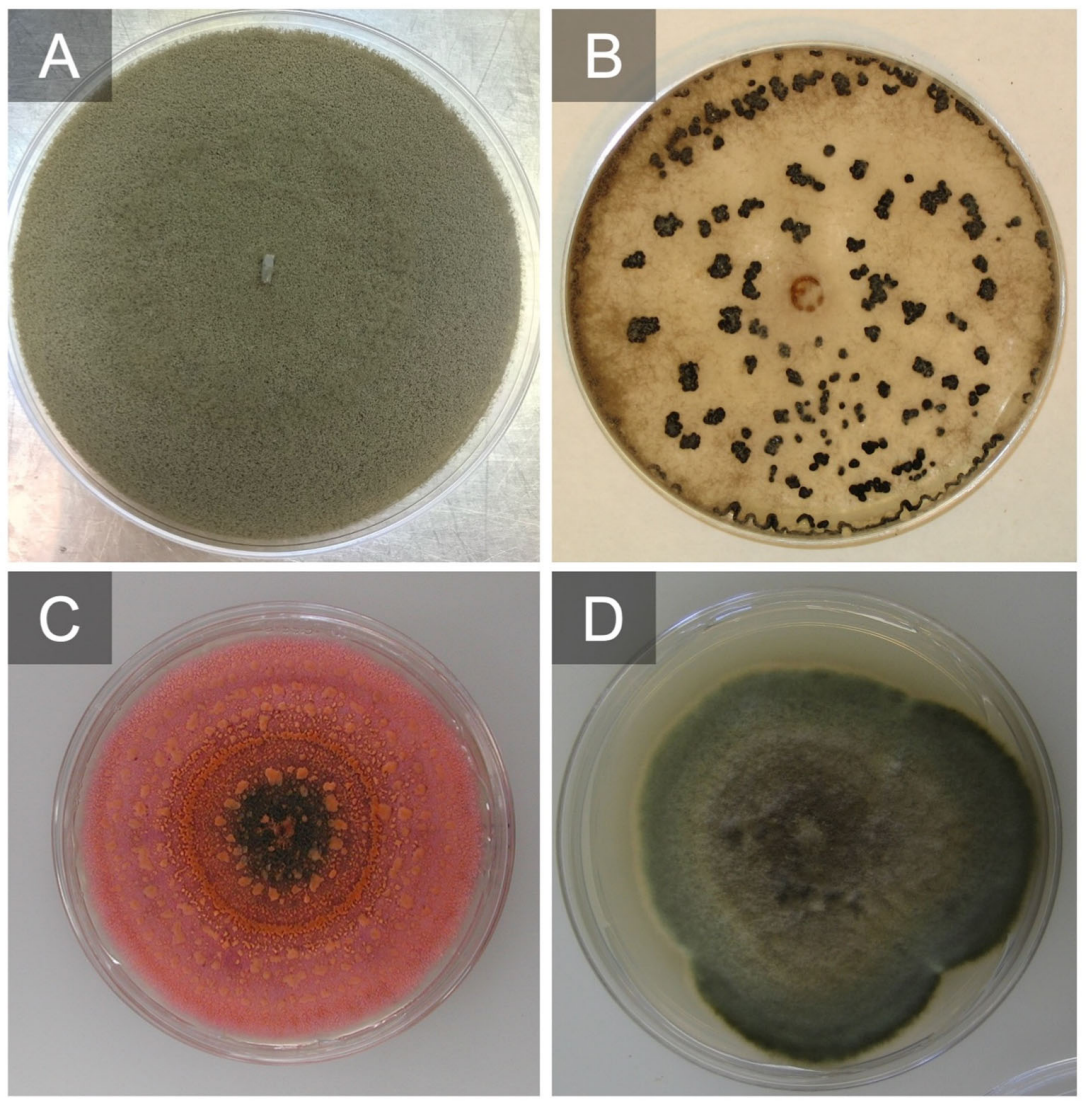
*Botrytis cinerea* grow on V8 agar producing conidia after 7 days **(A)** and grown on potato dextrose agar (PDA) producing sclerotia after 21 days **(B)**. *Colletotrichum fioriniae* culture producing conidia and a salmon-colored matrix after 10 days on PDA **(C)**. *Alternaria alternata* grown on PDA after 7 days **(D)**.

*Botrytis cinerea* is an opportunist pathogen, aggressively colonizing wounded or senescing host tissues (Bristow et al., 2017). *B. cinerea* overwinters as dormant mycelia or sclerotia (**Figure 4**) on plant debris (Richards et al., 2021; Adaskaveg et al., 2022). Masses of conidia produced in the spring from activated mycelia and germinated sclerotia are wind dispersed (Bristow et al., 2017; Adaskaveg et al., 2022, **Figure 4**). The first visual disease symptom of botrytis during a growing season is blossom blight (Adaskaveg et al., 2022, **Figure 1**). Flowers become susceptible just before opening but are most susceptible at full bloom. Under humid conditions, gray masses of conidia and mycelia can be seen on infected blossoms 3-4 days after infection (Bristow et al., 2017). Blossom blight can cause whole clusters of fruit to abort if infection is severe. From infected blossoms, disease will spread to twigs, leaves, and developing fruit. Blighted stems can become girdled and kill all tissue past the infection point (Bristow et al., 2017). As botrytis fruit rot is a polycyclic disease, new inoculum will infect the ovary and peduncle of developing fruit and lie dormant as the fruit matures. Fruit rot can occur preharvest, however, symptoms do not usually appear until after the fruit is harvested (Bristow et al., 2017). When harvested fruit is stored in humid and cool environments for long periods, the fruit will continue to rot and be covered by gray mycelia and masses of conidia (Adaskaveg et al., 2022, **Figure 3**). *B. cinerea* can grow in temperatures just above freezing, which makes it especially problematic during refrigerated storage and shipping (Bristow et al., 2017).

### 2.3 Infection mechanisms

**Figure 5:**
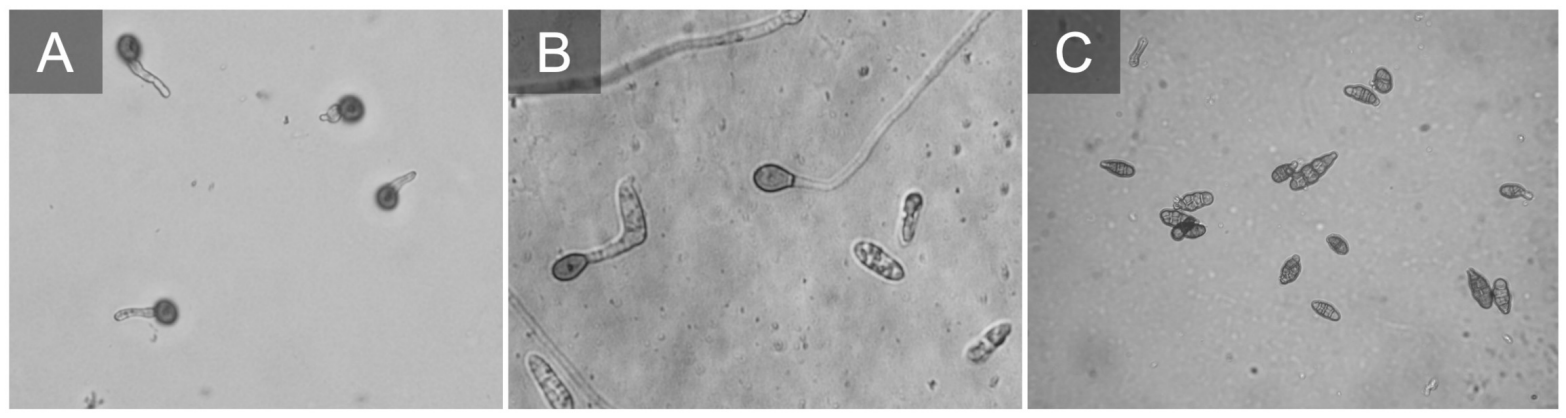
*Botrytis cinerea* conidia germinating **(A)**. *Colletotrichum fioriniae* conidia germinating and producing appressorium **(B)**. *Alternaria* sp. conidia typically on blueberries we observe small spores e.g. *Alternaria alternata* **(C)**.

Conidia are the primary inoculum of *B. cinerea* (Holz et al., 2004). A conidium germinates in free water or when the relative humidity is above 93% (Prins et al., 2000, **Figure 5A**). The germinating conidium secretes an extracellular matrix that aids in attachment and releases host cell wall degrading enzymes to facilitate penetrating the host (Prins et al., 2000; Kunz et al., 2006; Choquer et al., 2007). Appressorium-like structures are formed from conidia; however, they do not generate high amounts of pressure like a true appressorium (Choquer et al., 2007). *B. cinerea* can also generate penetration structures from mycelium, in which hyphae densely accumulate and swell at the tips to form a “claw-like” structure or “infection cushions” (Kunz et al., 2006; Choquer et al., 2007). After initial penetration, the pathogen kills the surrounding host tissue and establishes a primary lesion. As the lesion expands, more host tissue is macerated. The fungus colonizes the necrotic tissue and sporulates to produce inoculum for future infections (Prins et al., 2000).

## 3 Anthracnose fruit rot

### 3.1 Causal organism

*Colletotrichum* spp. cause destructive diseases, often referred to as anthracnose, on a variety of fruit crops including apple, peach, grape, blueberry, cranberry, and strawberry (Dowling et al., 2020). The two most common pathogenic species on blueberries are *C. acutatum* J.H. Simmonds and *C. gloeosporioides* Penz. & Sacc., which have both been resolved as species complexes based on multilocus sequencing (Damm et al., 2012; Weir et al., 2012). *C. gloeosporioides sensu lato* is the primary cause of ripe rot in the southeastern U.S (Adaskaveg et al., 2022). *C. gloeosporioides* spp. have also been reported to infect blossoms and stems of highbush blueberry (Dowling et al., 2020). Anthracnose fruit rot on blueberries in northern regions is most frequently caused by *C. acutatum sensu lato*, with the predominant species being *C. fioriniae* (Damm et al., 2012; Adaskaveg et al., 2022; Miles and Hancock, 2022). Anthracnose fruit rot was reported from all blueberry growing regions of North America as well as Australia, Japan, Korea, New Zealand, Norway, and Switzerland (Adaskaveg et al., 2022).

### 3.2 Lifecycle

**Figure 6:**
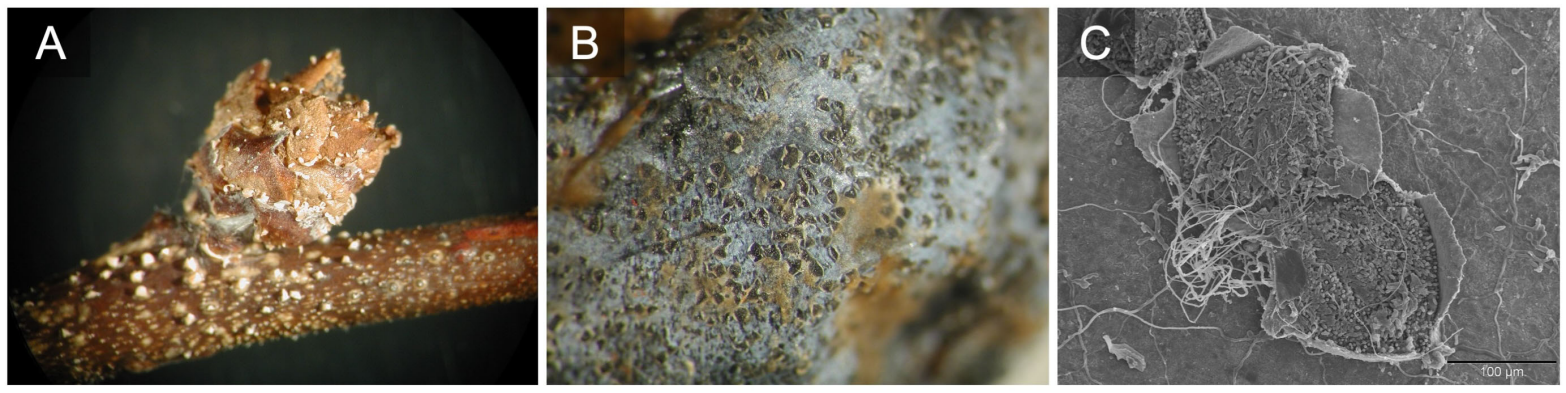
*Colletotrichum fioriniae* overwintering on blueberry bud and stem. Picture by Jerri Gillett **(A)**. Acervuli of *C. fioriniae* on fruit tissue after rinsing sporulating berries **(B)**. *C. fioriniae* acervuli under a scanning electron microscope **(C)**.

*Colletotrichum* spp. overwinter as mycelium on dead and dormant plant tissues (**Figure 6A**) (Milholland et al., 2017). Conidia are formed when temperatures exceed 11°C and are dispersed by rain droplets (Polashock et al., 2005; Verma et al., 2007; Miles et al., 2013; Milholland et al., 2017; Waller et al., 2018). Peak spore release occurs during the bloom period resulting in infections of blossoms, ovaries, and unripe fruit. Fatty acids released from the flowers increase spore germination and rate of infection making the bloom period particularly important for berry infection (Waller et al., 2023). The conidia germinate on the plant surface and attach via an appressorium (Polashock et al., 2005; Wharton and Schilder, 2008; Milholland et al., 2017, **Figure 5**). The appressorium becomes melanized, which allows increased turgor pressure to develop and penetrate the host epidermis (Miles et al., 2013). The resulting hyphae penetrate the epidermis but remain quiescent until the fruit ripens and physiological changes in the fruit trigger activation of the infection (Prusky and Lichter, 2007). As infection progresses, the ripe fruit soften around infection points and fruiting structures called acervuli (**Figures 6B, C**) break through the fruit surface producing droplets of orange/salmon-colored conidial masses (**Figure 1D**, **Figure 3B**). The conidia are water splash dispersed to cause secondary infection on adjacent fruit and other plant tissues, leading to stem dieback and blossom blight (Verma et al., 2007; Miles et al., 2013). The fungus will overwinter on vegetative tissues to serve as a source of inoculum the following year (DeMarsay, 2005; Waller et al., 2018).

### 3.3 Infection mechanisms

Initial infection development is essentially the same for all *Colletotrichum* species but after appressorium penetration of the host tissue, differences can be observed in infection strategies depending on the tissue or host being colonized (Wharton and Schilder, 2008). Generally, *Colletotrichum* species utilize either intracellular hemibiotrophy or subcuticular intramural necrotrophy (Perfect et al., 1999). In intracellular hemibiotrophy, a primary infection structure penetrates the host, which then colonizes the host tissue with primary and secondary hyphae. The infection process results in necrosis, but only late in the infection process. In subcuticular intramural necrotrophy, the fungus grows under the cuticle and produces thin necrotrophic hyphae that do not invade the host intracellularly (Miles and Schilder, 2013). In some hosts, such as strawberries, this differentiation of infection strategies is determined by the host tissue type; however, in blueberries the type of infection strategy seems to be determined by host resistance. In compatible interactions with the susceptible blueberry cultivar ‘Jersey’, *C. fioriniae* adopts an intracellular hemibiotrophic-like infection strategy but utilizes a subcuticular intramural infection strategy in incompatible interactions with the resistant cultivar ‘Elliott’ (Wharton and Schilder, 2008). With the susceptible cultivar ‘Jersey’, there was a higher rate of appressorium formation and intracellular infection structures were observed that facilitated the spread of the fungus (Miles et al., 2011). However, in the resistant cultivar ‘Elliott’, a lower rate of appressorium formation was observed, but appressorium did not penetrate the host cell wall and the fungus spread to neighboring cells via subcuticular hyphae (Wharton and Schilder, 2008; Miles et al., 2011).

## 4 Other blueberry fruit rots

### 4.1 Alternaria fruit rot

Alternaria fruit rot, caused by *Alternaria* spp., has been reported in most major blueberry production regions worldwide. While fruit rot symptoms can occur in season, most of the economic losses occur postharvest and in storage (Adaskaveg et al., 2022). Contaminated processing machinery and belts increase the incidence of alternaria fruit rot postharvest (Milholland and Cline, 2017). Alternaria fruit rot is more problematic in the Pacific Northwest region and California than other U.S. growing regions (Adaskaveg et al., 2022; Wang et al., 2022). *Alternaria* spp. can produce mycotoxins that can spread to healthy fruit tissue, even in the absence of visible fungal growth, which poses a health risk to consumers (Adaskaveg et al., 2022).

*Alternaria* spp. overwinter as conidia or mycelia in dry fruit or dead plant tissue. It rapidly colonizes injured or dead tissues producing conidia (**Figure 5C**). Spores are disseminated by wind or rain and mostly infect the lower leaves of the bush as light brown lesions surrounded by a red border. Defoliation occurs only in rare instances with severe infection and optimal environmental conditions (Milholland and Cline, 2017; Adaskaveg et al., 2022). Fruit infections start during bloom, but the infection is often latent until postharvest (**Figure 3C**). Rotted fruit is shriveled and covered with dark green-black mycelium and spores, often on the stem end of the berry (Milholland and Cline, 2017, **Figures 1E, F**, **3C**). During fruit storage, the ideal temperature for infection is 20°C, but infection can also persist at refrigeration temperatures (Adaskaveg et al., 2022). Storing and handling wet fruit can also increase disease incidence and spread (Milholland and Cline, 2017). *A. tenuissima, A. alternata*, and *A. arborescens* are the main *Alternaria* spp. that cause alternaria fruit rot of blueberry (Greco et al., 2012); however, there is still debate about the single primary species (Zhu and Xiao, 2015; Xiao and Saito, 2017; Adaskaveg et al., 2022; Wang et al., 2022).

Like other fruit rots, preventative control measures are necessary to control alternaria fruit rot. A fungicide spray program starting at pink bud through harvest will prevent blossom and fruit infections. Fungicides in Fungicide Resistance Action Committee (FRAC) codes 7, 9, 11, and 12 are effective at controlling alternaria fruit rot. Cultural practices, such as pruning bushes to ensure adequate spray coverage and to reduce overwintering inoculum, can also help reduce disease. Additionally, ensuring fruit is cooled rapidly and dry postharvest can limit losses in storage (Miles, 2020). The most resistant northern highbush cultivars are ‘Brigitta’, ‘Aurora’, ‘Elliott’, and ‘Draper’ (Retamales and Hancock, 2018).

### 4.2 Exobasidium fruit spot

Exobasidium fruit spot, first reported in 1998 in North Carolina (Cline, 1998), is limited to the southeast growing region on rabbiteye and southern highbush blueberry (Ingram et al., 2017). The disease can cause significant economic losses when infected fruits become unmarketable. In addition, yield losses due to defoliation and premature fruit drop can be significant when disease pressure is high (Brewer et al., 2014; Ingram et al., 2017; Ingram et al., 2019). The causal pathogen was initially identified as *Exobasidium vaccinii* (Cline, 1998) but was later determined to be *Exobasidium maculosum* through in-depth phylogenetic analysis (Brewer et al., 2014).

There is a limited knowledge about the epidemiology of exobasidium fruit spot of blueberry but there have been efforts to investigate a disease cycle (Cline and Brewer, 2017; Ingram et al., 2019). *E. maculosum* overwinters epiphytically on dead tissue as a saprophyte in a yeast-like conidial stage (Ingram et al., 2017; Ingram et al., 2019). In the spring, the yeast-like conidia are dispersed by splashing to infect newly emerging leaves and young green fruit. Leaf lesions are white/yellow and gradually turn necrotic while fruit spots are light green. The wax layer on the fruit may need to be removed to see initial symptoms. Fruit symptoms become more apparent as the fruit ripens and green spots become more visible (Ingram et al., 2017). The pathogen also causes necrotic lesions on young shoots that can girdle the stem (Ingram et al., 2017). *E. maculosum* produces basidiospores as disease progresses. However, it is proposed that basidiospores don’t play a role in reinfection during the growing season and mostly contributes to enabling sexual recombination and promoting dispersal to establish overwintering inoculum (Ingram et al., 2019). *E. maculosum* favors young tissue and extended wet periods of consecutive days with >1 mm rainfall (Ingram et al., 2017; Adaskaveg et al., 2022). Exobasidium fruit spot can be controlled with a late-dormant application of liquid lime sulfur to reduce the initial overwintering inoculum (Brannen et al., 2017).

### 4.3 Minor blueberry fruit rots

Other fungi that can cause fruit rots include *Phomopsis vaccinii, Pestalotia vaccinii, Phyllosticta vaccinii*, and unidentified yeast species. *Phomopsis vaccinii* (teleomorph: *Diaporthe vaccinii*) causes fruit to ripen prematurely and severe twig cankers that can lead to stem death. Infected fruit are characterized initially by white mycelia on the calyx and cream-colored spore droplets as infection progresses (Adaskaveg et al., 2022). *Pestalotia vaccinii* is a sporadic postharvest rot that forms black spore masses surrounded by white mycelia (Wharton and Schilder, 2008). *Phyllosticta vaccinii* causes leaf spot and fruit rot characterized by gray sunken fruit lesions (Adaskaveg et al., 2022). Yeasts occur sporadically when overripe fruit is harvested wet and can cause fruit to rapidly collapse and have a wet and slimy appearance on the berry. Yeast growth can exhibit white or pink slime, or black bumps (Wharton and Schilder, 2008; Adaskaveg et al., 2022).

## 5 Management

**Figure 7:**
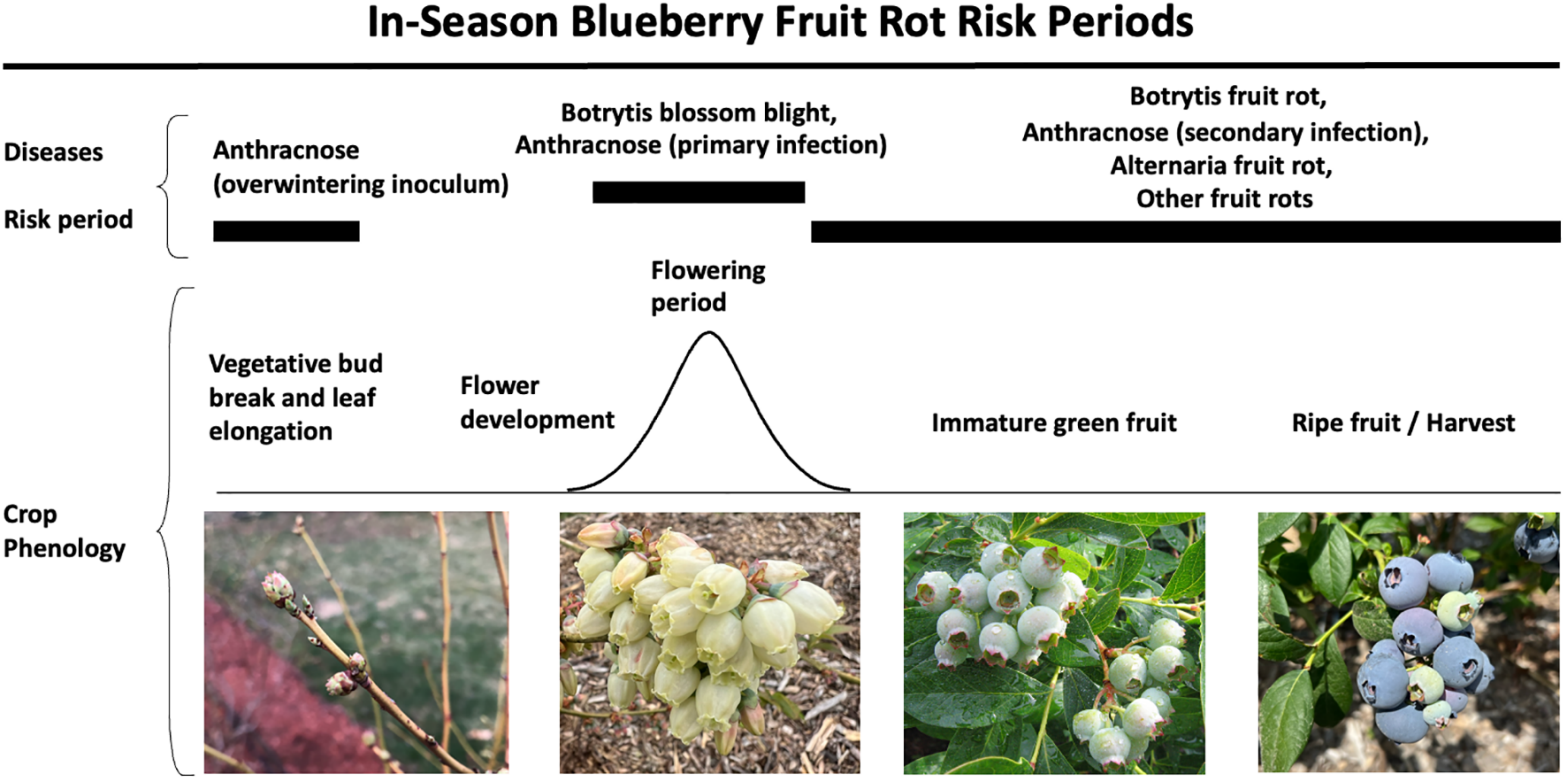
Risk periods for blueberry fruit rot diseases, including botrytis, anthracnose, alternaria, and other minor rots during the growing season. This figure highlights the risk periods and crop phenology associated with each disease.

Blueberry fruit rots are managed in two critical phases: pre- and post-harvest (**Figure 7**). Fungicides are the predominate method for pre-harvest management, but cultural practices can also be used (Stretch, 1967; Milholland et al., 2017; Adaskaveg et al., 2022; Wise et al., 2020). Post-harvest management is implemented through careful handling, sanitation, climate control, and use of antifungal volatiles or coatings. Although pre-harvest losses can be significant, postharvest development of these fruit rot diseases can lead to load rejections, reduction in shelf life, and dissatisfaction among consumers. As infections can be latent, high levels of pre-harvest infections lead to significant post-harvest losses. In the field, infections begin at the flowering stage and continue after fruit ripen (**Figure 7**). Inoculum from symptomatic fruit can contaminate healthy fruit and result in significant post-harvest loss under conducive conditions.

### 5.1 Cultivars

Blueberry cultivars vary in their resistance to anthracnose fruit rot (Retamales and Hancock, 2018). Most historically popular northern highbush blueberry cultivars are susceptible to anthracnose fruit rot, including ‘Berkeley’, ‘Bluecrop’, ‘Bluegold’, ‘Jersey’, ‘Nelson’, and ‘Ozarkblue’, but many of the recently developed northern highbush cultivars have anthracnose resistance, such as ‘Draper’, ‘Elliott’, ‘Legacy’, and ‘Little Giant’ (Polashock et al., 2005). In previous studies, resistance in specific cultivars to fruit infection by *Colletotrichum* was not correlated with foliar infection (Ehlenfeldt et al., 2006) or with antimicrobial fruit profiles (Polashock et al., 2007). Additionally, architectural resistance can play a role in fruit rot susceptibility. For example, DeMarsay (2005) demonstrated a relationship of bud scale drop with cultivar resistance. Furthermore, Waller et al. (2018) found wax extracts from flowers of some cultivars to be less stimulatory than other more susceptible varieties. Little is known about the resistance of highbush cultivars to botrytis fruit rot (Retamales and Hancock, 2018). Stem lesions caused by *C. gloeosporioides* have been evaluated (Phillips et al., 2018), but there has not been a formal study investigating susceptibility to fruit rots in southern highbush blueberry cultivars.

### 5.2 Cultural control

Traditional highbush blueberry in-row plant spacing has varied from 1.2 to 1.35 m (Moore et al., 1993). Over the past two decades, producers have been gradually transitioning to a closer in-row plant spacing of 1 m or less due to the substantial improvement in yield per acre (Moore et al., 1993; Strik and Yarborough, 2005). It could be argued that by decreasing in-row plant spacing increases plant canopy density, lowering fungicide efficacy, and exacerbating fruit rot incidence and severity. However, research showed that decreasing plant spacing lowered individual plant size, which improved canopy light interception (Bryla and Strik, 2007). Fruit exposure to sunlight decreases the incidence and severity of fruit rots across fruit crops (VanderWeide et al., 2020). Recent research suggests that the flavanol class of polyphenols, which are primarily located in fruit exocarp, improved resistance to fruit rots (Jacobs et al., 2023). Flavanol biosynthesis is stimulated in fruit by exposure to solar radiation (VanderWeide et al., 2022), and so an increase in canopy light interception may subsequently improve resistance to fruit rots.

Changes to irrigation practices may also favor better management of fruit rots in blueberry. Traditionally, blueberry plantings were established with overhead irrigation, particularly in growing regions with a higher risk for bud damage due to cold winter temperatures or spring frosts (Strik and Yarborough, 2005; Smith, 2019). Despite some benefits, the use of overhead irrigation during the growing season maintains canopy wetness, which promotes fruit rot development. Drip irrigation has been shown to maximize plant growth and irrigation water use efficiency in blueberry compared to other methods (Bryla et al., 2011) and is becoming the industry standard in many regions. This change in irrigation practice will decrease fruit rot incidence and severity in these plantings, as well as potentially lower fungicide use.

Harvest technique and timing can affect fruit rot development as well as post-harvest losses. The abrasive nature of mechanical harvesting leads to increased bruising and cuts on berries, which shorten their postharvest shelf life (Brown et al., 1996). Despite the implications of mechanical harvesting to exacerbate fruit rot severity, one study found no impact of mechanical versus hand harvesting on alternaria fruit rot, botrytis fruit rot, or anthracnose fruit rot development in four cultivars (Mehra et al., 2013). Frequent and adequate sanitization of harvesting machinery is crucial to eliminate microbes that may compromise food safety (Holland et al., 2022). The impact of mechanical harvesting on fruit rot development has not been evaluated; however, we can speculate that improper sanitization will spread fruit rot inoculum and infect healthy berries, which may reduce post-harvest shelf life. The degree of fruit ripeness at harvest may influence fruit rot development at both pre- and post-harvest. Botrytis fruit rot was more common in early harvests, while anthracnose and alternaria fruit rots were more frequent in late harvests (Cappellini et al., 1982). After blueberries ripen, changes still occur to berry composition, the most prominent of which are decreases in fruit firmness and organic acid concentration (Lobos et al., 2014). Firm berries tend to be more resistant to fruit rots (Mehra et al., 2013). It was found that organic acid concentration in berries negatively impacted *B. cinerea* and *A. alternata* fungal growth on media, while sugar concentration did not (Ballinger and Kushman, 1970). This suggests that shorter harvest intervals, particularly in cultivars without high fruit firmness or high organic acid concentrations, may decrease the risk for pre– and post-harvest fruit rot development.

### 5.3 Chemical control

In commercial production, growers typically rely on the use of fungicides for control of diseases caused by *Colletotrichum* and *Botrytis* spp. In addition to proper timing, fungicide efficacy is dependent on selection of effective fungicides, proper sprayer calibration, sprayer type, and pruning for coverage of the susceptible tissues (Hanson et al., 2000; VanEe et al., 2000). The expenses associated with control of these pathogens can be significant. For example, global expenses for fungicides targeting botrytis can exceed over $1 billion annually, which is about 10% of the fungicide market (Dean et al., 2012). For managing fruit rot diseases in blueberry production, fungicide applications largely focus on the period from bloom through harvest. Dormant applications of products containing lime sulfur, copper, or sulfur may also reduce disease carryover and thereby the incidence of these diseases (Bristow et al., 2017; Milholland et al., 2017).

Bloom is considered especially important for anthracnose control as it correlates with a peak in spore production by *Colletotrichum* (Polashock et al., 2005, **Figure 7**). DeMarsay (2005) demonstrated that a significant level of primary inoculum is contained within the outer bud scales of the overwintering inflorescence buds. However, whether the early-season inoculum or latent infection leads to fruit rot later in the season remains uninvestigated. In wine grapes, only late-season infections correlate with ripe rot (caused by *Colletotrichum* spp.) at harvest (Cosseboom and Hu, 2022). Preharvest management of blueberry anthracnose relies on well-timed applications of effective fungicides. Waxes released from developing flowers induce spore formation, rapid spore germination, and infection from host tissues (Waller et al., 2018). Protectant fungicides applied during the bloom period reduce infection levels on the developing ovaries. Once the outer bud scales have dropped, only the successful infections of the plant tissues remain behind. These latent infections may become active with the onset of tissue senescence and fruit ripening. Any type of damage ranging from phytotoxic effects of pesticides, overheating of plant tissues, hail injury, or drought damage facilitates the activation of the pathogen. Thus, well-timed fungicide applications targeting initial inoculum could be the first critical step in controlling the disease. Likewise, for botrytis control, bloom time is the most important period due to economic losses resulting from blossom blight. Though fruit that is set is generally considered to be resistant, infected corollas provide an entryway for spread of botrytis to fruit (Sciarappa and Oudemans, 2005).

Nonetheless, additional applications during fruit development are needed to prevent fruit infection, especially during wet seasons, and recommendations for control of botrytis and anthracnose fruit rot suggest that applications start at pink bud or early bloom and continue every 7-10 days through the preharvest period can be effective (Wise et al., 2022; Sial et al., 2023). Along with other materials (**Table 1**), active ingredients frequently utilized for botrytis control in blueberries include chemicals in FRAC classes 7, 9, 11, 12, and 17, while chemicals in FRAC groups 3, 9, 11, and 12 are typically recommended for anthracnose control (Beckerman et al., 2022; Wise et al., 2022; Sial et al., 2023). Demethylation inhibitors (DMIs; FRAC 3) are variable in their performance against *Colletotrichum* spp. and are generally not recommended for anthracnose control unless prior experience indicates the efficacy of a particular member of this fungicide class (Milholland et al., 2017; Sial et al., 2023). Multisite fungicides including ziram (FRAC M3), captan (FRAC M4), and chlorothalonil (FRAC M5) are also utilized for control of botrytis and anthracnose on blueberry and can be an important fungicide resistance management tool (Sial et al., 2023). New fungicides, including FRAC 7 materials, are also being developed for anthracnose control.

**Table 1:** Fungicide active ingredients labelled for use in blueberry effective against anthracnose fruit rot and/or botrytis fruit rot (FRAC, 2024; Telus Agronomy Label Database, 2024; Wise et al., 2022).

Fungicide MOA	FRAC	Active Ingredient
G1: sterol biosynthesis in membranes	3	metconazole, fenbuconazole, propiconazole, prothioconazole, difenoconazole
C2: succinate dehydrogenase inhibitors	7	boscalid, fluopyram, isofetamid, pydiflumetofen, penthiopyrad
D1: methionine biosynthesis inhibitor	9	cyprodinil, pyrimethanil
C3: quinone outside inhibitors	11	azoxystrobin, pyraclostrobin
E2: osmotic signal transduction	12	fludioxonil
G3: keto-reductase inhibitor	17	fenhexamid
C4: quinone inside inhibitor	21	florylpicoxamid**
C5: uncouplers of oxidative phosphorylation	29	fluazinam
P07: phosphonates	P 07	fosetyl-Al, phosphorous acid and salts
M: multisite fungicides	M	copper (M1), ziram (M3), captan (M4), chlorothalonil (M5), folpet (M4)*

*not registered in the U.S., but in other locations.

**pending registration in the U.S. in blueberries.

### 5.4 Biopesticides

With increasing levels of fungicide resistance in pathogen populations, concerns about chemical residues on fruit at export, and increasing awareness of pollinator protection during bloom, there has been a need for alternatives to conventional chemical fungicides to control fruit rots. Biopesticides are naturally occurring substances and/or microorganisms such as bacteria, fungi, or yeasts, that aim to reduce the incidence of fruit rot using one or more mechanisms (Environmental Protection Agency, 2023). Some common mechanisms among biopesticides products include creating competition for space and nutrients, antibiosis, and induction of host plant resistance (Abbey et al., 2019; Bell et al., 2021; Turc et al., 2022). Antibiosis is a common mode of action for certain bacterial biopesticides. For example, some strains of *Pseudomonas* have been shown to produce pyrrolnitrin, an antibiotic that inhibits mycelial growth of *B. cinerea* (Ajouz et al., 2010; Loper et al., 2012) and various strains of *B. subtilis* produce broad spectrum antibiotic substances, antifungal secondary metabolites (cyclic lipopeptides), and lytic enzymes (chitinases) (Leifert et al., 1995; Emmert and Handelsman, 1999; Stein, 2005; Lin et al., 2021). Yeasts do not produce as many secondary metabolites as bacteria and little is known about their specific mode of action as biopesticides, thus competition is considered the main mode of action. However, competition is efficient for *B. cinerea* control as conidial germination and germ tube growth are nutrient dependent (Roca-Couso et al., 2021). Naturally occurring substances such as essential oils and plant extracts are also used as biopesticides to reduce pathogen growth and disease incidence. It is thought that the hydrophobic nature of essential oils enable accumulation in the cytoplasmic cell membrane of the pathogen, which disrupts cell structure and increases permeability (Abbey et al., 2019).

There are several biopesticides commercial products that are labeled for use on blueberry for fruit rot management listed in **Table 2**. *Bacillus subtilis*, the microbe in Serenade ASO, has been reported to produce lipopeptides that disrupt fungal cell membranes and induce host plant internal defenses related to PR1 upon contact (Ongena et al., 2010). *Streptomyces lydicus*, the microbe in Actinovate, provides efficacy through a combination of competitive exclusion and secretion of chitinases, glucanases, and peroxidases that target fungal walls and membranes (Crawford et al., 2005; Lichatowich, 2007). The two yeast strains of *Aureobasidium pullulans* that make up Botector also act through competitive exclusion, limiting nutrients and colonization space (Schilder, 2013), although recently these yeasts were shown to induce PR gene expression and the accumulation of salicylic acid in pome fruit flowers (Zeng et al., 2023). Tea tree oil (Timorex ACT) has been shown to inhibit conidial germination and mycelial growth of *B. cinerea* (Nicot et al., 2015) and has been reported to alter the morphology of *B. cinerea* mitochondria (Li et al., 2017). An extract of *Reynoutria sachalinensis* (giant knotweed), used in Regalia, triggered induced systemic resistance in the host plant to inhibit *B. cinerea* (Nicot et al., 2015).

**Table 2:** Biopesticides labelled for use in blueberry for management of anthracnose fruit rot and/or botrytis fruit rot.

Mode of Action	Active Ingredient	Product Name
Competitive Exclusion	*Bacillus amyloliquefaciens* D747	Double Nickel (Certis) (Certis Biologicals, 2024)
Antibiosis	Mixture of lipopeptides synthesized by *Bacillus subtilis* QST 713	Serenade ASO (Bayer) (Ongena et al., 2010)
Competitive Exclusion	*Aureobasidium pullulans* strain DSM 14941 and DSM 14940	Botector (SAN Group Biotech) (Schilder, 2013; Nicot et al., 2015)
Competitive Exclusion	*Bacillus amyloliquefaciens* F727	Stargus (Marrone Bio) (Marrone Bio Innovations, 2024)
Competitive Exclusion	*Bacillus amyloliquefaciens* MBI 600	Serifel Biofungicide (BASF) (BASF, 2024)
Competitive Exclusion	*Streptomyces lydicus* WYEC108	Actinovate (Novozymes BioAg) (Crawford et al., 2005; Lichatowich, 2007)
Fungicidal	Tea tree oil	Timorex ACT (Summit Agro) (Nicot et al., 2015)
Fungicidal	Garlic and cinnamon oil	Gargoil (SAN Group Biotech) (Abbey et al., 2019)
Induce systemic resistance of host plant	Extract of *Reynoutria sachalinensis*	Regalia (Marrone Bio) (Nicot et al., 2015)
Induce systemic resistance of host plant	*Bacillus mycoides* isolate J	Lifegard (Certis) (Certis Biologicals, 2024)
Chitin inhibitor	Polyoxin D zinc salt	Oso (Certis) (Certis Biologicals, 2024)

Table modified from Nicot et al., 2015.

Biopesticides may be integrated with synthetic fungicides for an integrated disease management program, but the label should be examined carefully for potential incompatibilities (Fravel et al., 2005). For example, according to the Botector product label, the two strains of *A. pullulans* are sensitive to many synthetic fungicides and copper-based products. In contrast, the biopesticide Serenade (*Bacillus subtilis* QST713) can be tank mixed with synthetic fungicides according to the product label. Timing of application of biopesticides is another consideration for a spray program. A product whose mode of action is competitive exclusion, such as Botector, should be applied prior to fungal colonization and infection periods to provide time for the yeasts to establish large populations in order to exclude the pathogen from infection sites (Pertot et al., 2017). Similarly, biopesticides whose mode of action is centered on induction of plant defense mechanisms should be applied early enough to activate those pathways prior to establishment of the pathogen. Unfavorable environmental conditions during or after application of biopesticides such as cold temperatures or excessive rain also can lead to failure of the organism to establish and colonize tissues (Nuclo et al., 1997; Altieri et al., 2023). Overall, integration of biopesticides into a disease management program, especially at critical stages of fruit development, such as bloom and near harvest, may reduce the number of synthetic fungicide applications required, reduce fungicide residues on berries, decrease selection pressure for fungicide resistance, and improve fruit rot control.

### 5.5 Post-harvest

Despite the many cultural and chemical practices that impact pre-harvest fruit rot development, fruit rots largely develop during post-harvest storage (Caruso and Ramsdell, 1995), suggesting the importance of post-harvest conditions to slow and minimize development. The development of fruit rots is rapid at room temperature; refrigeration at 10°C was effective at lowering disease incidence (Cappellini et al., 1972). Furthermore, storage at 0°C significantly decreased fruit rot incidence compared to 4°C (Borecka and Pliszka, 1985; Paniagua et al., 2014). In general, storage temperatures near 0°C suppress fruit rot development and prolong shelf life. Use of extended controlled atmosphere storage (high levels of carbon dioxide) can suppress fruit rots but is cultivar dependent and can result in negative effects on fruit quality such as internal discoloration and reduction in fruit firmness (Alsmairat et al., 2011). Post-harvest fumigation with sulfur dioxide is frequently used to control botrytis in table grapes (Luvisi et al., 1992) and research has shown it to be effective in blueberries as well (Cantin et al., 2012). Calcium or chitosan based edible coatings have also been evaluated and can help reduce the decay rate during storage (Duan et al., 2011). Hot water treatments were effective at reducing rot caused by *B. cinerea* and *C. fioriniae* but did reduce titratable acidity and soluble solids (Fan et al., 2008). Not only do cultivars ‘Aurora’, ‘Draper’, and ‘Brigitta’ have resistance to anthracnose, but they were found to have longer storage life than ‘Duke’, ‘Bluecrop’, ‘Jersey’, and ‘Elliott’ (Hancock et al., 2008).

## 6 Diagnostics

### 6.1 Pathogen detection

Pathogen detection is foundational to design appropriate management strategies. Blossom blight and stem dieback symptoms can be caused by *Botrytis* spp. and *Colletotrichum* spp., but these symptoms can also be caused by a wide variety of biotic and abiotic factors, such as *Diaporthe* spp.*, Pseudomonas syringae*, or frost damage (**Figure 2**). Incubating affected host material in a humid chamber followed by observing sporulating structures under a dissecting microscope can serve as the first line to detect and identify the causal agent. However, this approach is complicated to differentiate species within a fungal genus that are morphologically similar but possess different levels of sensitivity to fungicides, which in turn dictates management approaches. For example, both *B. cinerea* and *B. pseudocinerea* can infect blueberries and exhibit inherent differences in their resistance response to fenhexamid fungicide but are difficult to differentiate using morphological features. Hence, a polymerase chain reaction (PCR)-based assay was developed to differentiate *B. cinerea* from *B. pseudocinerea* based on a 24-bp indel region in the *BC1G_07159* gene (Plesken et al., 2015). Likewise, a multiplex PCR assay to detect and differentiate *B. cinerea* from its closest related species *B. fabae* from infected broad bean samples was developed by Fan et al. (2015). Several such molecular assays developed to identify pathogens to species level are shown in **Table 3**.

**Table 3:** Molecular markers for *Botrytis* or *Colletotrichum* species that have utility in blueberries.

Pathogen	Type of Assay	Target Locus	Purpose	Reference
*B.* spp	PCR	ITS1 and ITS4	Species identification	White et al., 1990
*B.* spp	PCR	*Flipper*	Separate Group I vs II	Levis et al., 1997
*B.* spp	PCR	*Boty*	Separate Group I vs II	Diolez et al., 1995
*B. cinerea*	TaqMan qPCR	B-tubulin, IGS, SCAR	*B. cinerea* detection	Suarez et al., 2005
*B. cinerea*	SYBR qPCR	IGS	*B. cinerea* detection	Diguta et al., 2010
*B. cinerea*	LAMP	*Bcos5*	*B. cinerea* detection	Duan et al., 2014
*B.* spp	PCR RFLP	Bc*-hch*	*B. pseudocinerea* detection	Fournier et al., 2003
*B.* spp	PCR	*MS547*	*B. cinerea* vs *B. pseudocinerea*	Walker et al., 2011
*B.* spp	PCR	*G3PDH, HSP60, RPB2*	Molecular phylogeny	Staats et al., 2005
*B.* spp	Microsatellite markers	Various	Population diversity	DeLong et al., 2020
*B. cinerea*	TaqMan qPCR	*erg27*	Fenhexamid resistance	Alzohairy et al., 2021
*B. cinerea*	TaqMan qPCR, rhAMP	*sdhB*	Boscalid and fluopyram resistance	Alzohairy et al., 2023
*B. cinerea*	HRM	*sdhB*	SDHI resistance	Samaras et al., 2016
*B. cinerea*	LAMP	*sdbB*	Boscalid resistance	Fan et al., 2018
*B. cinerea*	Suspension array multiplex PCR	*BenA, SdhB, BcOS1*, and *erg27*	Benzimidazole, boscalid, dicarboximide, and fenhexamid resistance	Zhang et al., 2016a
*C. acutatum* SC	PCR	ITS*, ACT, TUB2, CHS-1, GAPDH, HIS3*	Differentiate *C. acutatum* species complex	Damm et al., 2012
*C. gloeosporioides* SC	PCR	ITS	Differentiat*e C. gloeosporioides* species complex	Weir et al., 2012
*C. acutatum* SC	TaqMan qPCR	ITS1	Detect *C. acutatum* species complex	Debode et al., 2009; Martin and Peter, 2021

Quantitative real-time PCR (qPCR) assays have also been developed to detect and quantify *B. cinerea* in a range of host species. The internal transcribed spacers (ITS1 and ITS2) are commonly used to identify fungi at the species level. However, in *Botrytis*, the ITS regions have very few sequence differences among them, making a species-specific assay difficult. Suarez et al. (2005) developed a qPCR assay based on hydrolysis probes targeting the intergenic spacer (IGS) region between 28S and 18S genes on the nuclear ribosomal DNA that was highly specific to *B. cinerea.* The presence of plant extracts did not interfere in these assays and DNA of *B. cinerea* as low as 20 fg could be detected, thus enabling detection of the pathogen at low titers in the plant material (Suarez et al., 2005). Cadle-Davidson (2008) developed another qPCR assay with a detection limit of 100 fg of *B. cinerea* DNA. Diguta et al. (2010) modified Suarez et al. (2005) primers for SYBR Green and utilized exogenous *Yarrowia lipolytica* DNA for an internal control. However, the limit of detection with this assay was determined to be 6.3 pg, which was lower than that described by Suarez et al. (2005). An EvaGreen (similar to SYBR Green) based qPCR assay was developed based on the *RPB2* gene by Moretti et al. (2015). However, the assay not only detected *B. cinerea* but also phylogenetically related species such as *B. fabae* and *Botryotinia pelargonii*. An alternative to PCR-based assays are isothermal assays, such as loop-mediated isothermal amplification (LAMP). Duan et al. (2014) developed a *B. cinerea* specific LAMP assay based on the *B. cinerea Bcos5* gene.

Likewise, a qPCR assay based on a hydrolysis probe was developed by Debode et al. (2009) and adapted by Martin and Peter (2021) to detect and quantify DNA of *C. acutatum* species complex. Similar to *Botrytis*, LAMP assays were also developed to detect asymptomatic infections caused by *C. acutatum* sensu lato (Zhang et al., 2016b).

### 6.2 Population genetics

The genus *Botrytis* is highly genetically diverse with more than 30 species that differ in morphology, ecology, biology, and host range, which makes population genetics complicated (Waller et al., 2018; Naegele et al., 2021). Various molecular markers have been developed to study the genetic variability and population structure and are described in **Table 3**. Staats et al. (2005) conducted molecular phylogenetic analyses of 22 *Botrytis* species based on the analysis of three genes encoding glyceraldehyde-3-phosphate dehydrogenase (*G3PDH*), heat shock protein 60 (*HSP60*), and DNA-dependent RNA polymerase subunit II (*RPB2*). This analysis supported two clades based on host range, one clade that mostly infects monocots and some dicots, while the second clade contained isolates that infect a wide range of eudicots. *B. cinerea* clustered in the second clade along with three other species (Staats et al., 2005). Fournier et al. (2003) developed a PCR-RFLP assay that differentiated *B. pseudocinerea* from the *B. cinerea* complex based on a detected polymorphism in the Bc-*hch* gene. *B. pseudocinerea*, formerly known as *B. cinerea* Group 1, is morphologically identical to *B. cinerea* and can occur on the same hosts (Fournier et al., 2005; Walker et al., 2011). DeLong et al. (2020) developed a set of nine microsatellite markers spanning the genome to characterize diversity among *B. cinerea* populations collected in California from three years. Significant pairwise differentiation was detected among years, and locations, but most of the diversity was observed within individual field subpopulations (DeLong et al., 2020). Similar localized populations with limited migration among adjacent fields was noted by Kozhar et al. (2020) using a set of seven microsatellite markers on *B. cinerea* populations from small fruit crops in the Pacific Northwest.

Unlike *Botrytis*, classification is much more complex for *Colletotrichum* species that cause blueberry fruit rots. Multiple gene sequences (actin, calmodulin, chitin synthase, glutamine synthetase, manganese superoxide dismutase, beta tubulin 2, ribosomal internal transcribed spacer, glyceraldehyde-3-phosphate dehydrogenase, histone3) were aligned to conduct phylogenetic analyses to differentiate *C. acutatum* and *C. gloeosporioides* species complexes (Damm et al., 2012; Weir et al., 2012). An attempt was made to determine a group of markers to distinguish all species of *Colletotrichum*, however it was unsuccessful. Some markers were effective for one complex, but were not effective for another (Vieira et al., 2020).

### 6.3 Fungicide resistance

A central question that growers constantly encounter while making fungicide applications is whether a particular product is effective in managing the target pathogen. In-season regimented spray schedules for fruit rot management invariably lead to pathogens developing resistance to different classes of fungicides. For example, *Botrytis* can develop resistance to multiple fungicide classes and the pathogen population is structured based on field level management practices such as fungicide applications (Kozhar et al., 2020). This warrants regular monitoring of the pathogen to determine field efficacy of fungicides. Scientific literature is replete with studies determining pathogen sensitivity to several fungicide chemistries using either spore germination assays or mycelial growth assays on fungicide amended agar media. However, these non-high throughput assays are time consuming and involve using a large amount of media and Petri plates. Furthermore, an isolate can be designated as resistant or sensitive to a particular fungicide chemistry based on the perception of the observer, which can impact grower management decisions.

An alternative approach for monitoring fungicide resistance is to use sensitive and specific molecular tools for detecting the diversity of mutations resulting in fungicide resistance as well as their differential effects on cross-resistance patterns. **Table 3** outlines molecular approaches such as LAMP, qPCR, and high-resolution melting (HRM) assays to detect mutations in target genes. For instance, resistance to QoI fungicides (FRAC 11) occur due to point mutations in the *cytb* gene (G143A, F129L, and G137A), which confer different levels of resistance. Similar point mutations in the succinate dehydrogenase subunit B confer different levels of resistant phenotypes in *B. cinerea* (Amiri et al., 2014). Likewise, several mutations in the *erg27* gene (F412S, G170R, A201G) and beta-tubulin gene (E198A) confer resistance to FRAC 17 group fungicides in *B. cinerea* and to FRAC 1 fungicides in *Colletotrichum* isolates, respectively (Amiri and Peres, 2014; Hu et al., 2015). These tools have improved rapid resistance risk assessment and deployment of appropriate resistance management strategies. However, each of these methods has its own set of limitations in terms of cost and ease of use. A major limiting factor associated with most of these assays is the need to run multiple reactions or reactions with multiple probes to identify diverse mutations associated with fungicide resistance. In this case, traditional bioassays may be more efficient and useful as they pick up resistant phenotypes regardless of genotypes or mutations involved.

Like many other pathosystems, fungicide resistance in blueberry fruit rot pathogens, particularly those driving the need for targeted sprays to manage botrytis fruit rot is not uncommon. In a 2012 and 2013 survey conducted from packinghouses in California, Saito et al. (2016) found that out of 249 *B. cinerea* isolates collected, 66% were resistant to boscalid, 66% were also resistant to pyraclostrobin, 29% were resistant to fenhexamid, and 20% were moderately resistant to cyprodinil. Similar frequencies also were detected in isolates from Washington, USA, with many displaying diverse fungicide-resistance phenotypes (Saito et al., 2016). Amiri et al. (2018) evaluated resistance in *Botrytis* isolates from blueberry fields grown in the vicinity of intensely managed strawberry fields or not in Florida, with 181 and 432 isolates collected from the two field types, respectively. Interestingly, resistance frequencies for all fungicides tested, including phenthiopyrad, cyprodinil, boscalid, fenhexamid, pyraclostrobin, and thiophanate-methyl, were always higher from isolates collected close to strawberry fields. In addition, isolates simultaneously resistant to six or five fungicide groups were found to be predominant (50 to 70%), regardless of the field type. This indicates that resistance selection may start at nearby cropping fields with commonalities in pathogens and spray materials (Amiri et al., 2018).

Resistance has also been reported to other blueberry fruit rot pathogens. A recent study evaluated fungicide resistance in 143 A*. alternata* isolates from California and all were determined to be resistant to boscalid, 42 were resistant to fluopyram, and 60 isolates were resistant to QoI fungicides (Wang et al., 2022). Furthermore, resistance was detected to both thiophanate-methyl and azoxystrobin in *Colletotrichum siamense* isolates from South Carolina (Hu et al., 2015) and similar resistant phenotypes were also found in Georgia, USA (Ali et al., 2019). These results highlight the importance of resistance management.

## 7 Future directions

### 7.1 Breeding for disease resistance

Like most perennial crops, breeding blueberries through traditional methods can take 10 years or more to release a new cultivar (Edger et al., 2022) Accelerating this process and emphasizing breeding for disease resistance to reduce fungicide use and increase fruit quality and yield are primary goals of researchers and industry (Edger et al., 2022). Utilizing genomic resources such as marker-assisted selection is one way to improve early trait selection and reduce overall breeding time. Miles and Hancock (2022) recently determined that anthracnose resistance is a quantitative heritable trait, and there are likely multiple loci involved in resistance. However, heritability of unique forms of resistance such as fatty acid profiles (Waller et al., 2023) or bud scale dehiscence (DeMarsay, 2005) are unknown. Quantitative resistance controlled by multiple loci has been reported in many *Colletotrichum* spp.-host interactions including strawberry (Geffroy et al., 2000; Denoyes-Rothan et al., 2005; Miles and Hancock, 2022). The underlying genetic mechanisms of anthracnose resistance in blueberry has not been exhaustively investigated (Jacobs et al., 2023). Future work to identify quantitative trait loci (QTLs) or single nucleotide polymorphism (SNPs) associated with resistance would be useful for developing a marker-assisted selection pipeline and advancing disease resistance breeding in blueberry. In a recent study, Jacobs et al. (2023) developed a ‘Draper’ x ‘Liberty’ mapping population with variation in susceptibility to anthracnose fruit rot and identified three chromosomes (17, 23, and 28) associated with anthracnose resistance. The candidate genes within the identified QTLs have been previously associated with pathogen resistance and flavonoid biosynthesis. The identification of these loci will be useful for developing a molecular marker-assisted selection protocol for blueberry, which will facilitate screening for anthracnose fruit rot resistance early in the breeding and selection process.

### 7.2 Engineering advances

The ever-growing demand for high-quality fruit and rising labor costs are driving engineering research and development in postharvest grading and sorting of blueberries. Blueberry sorting based on visual inspection and hand sorting is the traditional method used at most packing facilities. This type of manual grading and sorting is labor-intensive, less efficient, and often unreliable. Development and implementation of high throughput, automated systems are becoming imperative for blueberry industries to be sustainable and competitive. Sorting based on fruit firmness is important to producing fresh-market blueberries with mechanical harvesters becoming more widely used. Mechanically harvested fruit contains varying amounts of overripe or too soft berries (Dale et al., 1994), which are more susceptible to rot development during post-harvest storage than hand-harvested fruit. Separation of berries based on firmness is effective for reducing decay of machine-harvested blueberries (Wolfe et al., 1983). Tilt belts used in processing lines can separate very soft berries that do not roll as easily as firm fruit. However, removing soft berries by exploiting this difference in a combination of rolling and bouncing characteristics may be more efficient (Wolfe et al., 1983). Early attempts demonstrated the feasibility of sorting blueberries for firmness based on vibration responses (Hamann et al., 1973) and rebounding or impact force characteristics (e.g., peak impact force) (Rohrbach et al., 1982). These sorting concepts have been implemented in non-vision commercial sorting systems (e.g., “Inline Bounce Sorter” from A&B Packing Equipment Inc., Lawrence, MI, USA), but they do not provide direct quantification of fruit firmness levels.

Imaging technology is the most widely used for automated fruit grading and sorting according to appearance and quality attributes, such as size, color, and surface defects. Color imaging is well suited for online, high-speed quality inspection, while hyperspectral imaging is more powerful for assessing both external and internal quality characteristics of blueberries (Leiva-Valenzuela et al., 2014; Zhang et al., 2017), but is still restricted to laboratory use due to instrumentation, speed, and cost constraints in practice (Lu et al., 2020). Several imaging-based blueberry sorting systems are commercially available, e.g., “BerryTeck Sortivator” (WECO, Woodland, CA), Blueberry Vision 3 (Unitec, Lugo RA, Italy), and “KATO260” (TOMRA Food, Leuven, Belgium). These systems are generally equipped with high-resolution color cameras that acquire a sequence of different views of each blueberry rotating on a customized conveyor for full surface quality inspection. Commercial vision systems that employ near-infrared cameras for blueberry defect detection have recently become available, e.g., “Air Jet Blueberry Grader” (GP Graderes, Victoria, Austria) and “Green Sort” (Janów Podlaski, Poland). However, sorting for specific types of defects, such as fruit rots, is still a challenging task, especially when the identifying characteristics are not noticeable. As artificial intelligence (AI) is advancing and finding its utility in food processing in recent years, AI-powered sorting solutions are being investigated in research communities and industries, which are expected to enhance blueberry grading to meet precision market demands in the years to come. To develop effective systems for blueberry rot detection, considerable research remains to be done to evaluate emerging imaging modalities that are proven effective in detecting rots in other horticultural commodities (e.g., peaches, oranges), such as structured light imaging (Sun et al., 2019; Li et al., 2023, Li et al., 2024).

### 7.3 Harnessing chemical markers

The critical period of management for anthracnose control begins during bloom (Waller et al., 2018) because there is a synchronization of an increase in conidial production and bloom (Wharton et al., 2002; DeMarsay, 2005; Miles et al., 2013). Waller et al. demonstrated that water soluble extracts from blueberry flowers promoted and increased *C. fioriniae* germination, appressorium formation, and secondary conidiation (Waller et al., 2018). In a detached fruit assay, floral extracts added to *C. fioriniae* inoculum increased infection and symptom development (Waller et al., 2018). These compounds were also found to shorten the infection period and increase the temperature range infection could occur (Waller et al., 2023). There were two fatty acids identified in aqueous floral extracts, which demonstrated that these floral compounds can be water dispersed and could affect sporulation on other plant tissues (Waller et al., 2018). Floral extracts from resistant and susceptible cultivars were found to stimulate secondary conidial production; however, extracts from susceptible cultivars triggered more appressorial production than extracts from resistant cultivars (Waller et al., 2018). These compounds could be utilized in future breeding efforts to generate chemical markers for high-throughput phenotyping, used as a novel control practice, or monitored to improve the accuracy of epidemiological models.

### 7.4 Monitoring fungicide resistance for informed management decisions

This review covered aspects related to integrated disease management approaches for fruit rots in blueberries, which are quite reliant on spray programs involving use of several single-site and multi-site synthetic fungicides as well as how fruit rot pathogens have evolved to develop resistance to these fungicides. Researchers have been developing cultural and molecular assays to screen for resistance and identify genetic mechanisms that result in the development of fungicide resistance. However, improved disease management can be realized only when such developments are put into practice for grower adoption and acceptance. Research indicates that management practices taken on a field-scale or nearby fields shape populations of fruit rot causing pathogens such as *Botrytis* spp. in blueberries (Amiri et al., 2018; Kozhar et al., 2020). Growers are aware of fungicide resistance issues and are committed to judicious use of the limited tools in their toolbox to raise a profitable crop. From a growers’ lens, two persisting questions that need researchers’ attention are ‘which’ fungicides are still effective and ‘when’ to incorporate them in the spray program so their return on investment can be maximized. This warrants the need for developing standardized high-throughput fungicide sensitivity screening assays that are reliable and reproducible across laboratories. Availability of such testing facilities at growers’ disposition where results could be provided within a short turnaround time for making in-season management decisions will not only improve on-farm efficiency but also promote fungicide stewardship.

### 7.5 Economic drivers for controlling fruit rots

There are several economic ramifications of blueberry fruit rot mitigation strategies. The issues include rising input prices, increased regulation of fungicides, and limited availability of new fungicides (Brumfield and Pavlis, 2019; Beckerman et al., 2023). Budget analyses are needed to investigate the implications of reduced fungicide efficacy (due to either the presence of fungicide resistance or a less strong fungicide) and the adoption of new strategies, considering rising input prices, regulatory constraints, and limited availability of new fungicide active ingredients. Additionally, a budget analysis could be shared with other blueberry growers to assess the impact of biologically based pesticides on input costs and production management, potentially leading to a change in application frequency within a growing season. The analysis could provide insights into the profitability of rot-resistant cultivars and alternative disease control measures (e.g. pruning strategies, harvest method), informing decision-making for managing fruit rot across different levels.

### 7.6 Shared learning of management

**Figure 8:**
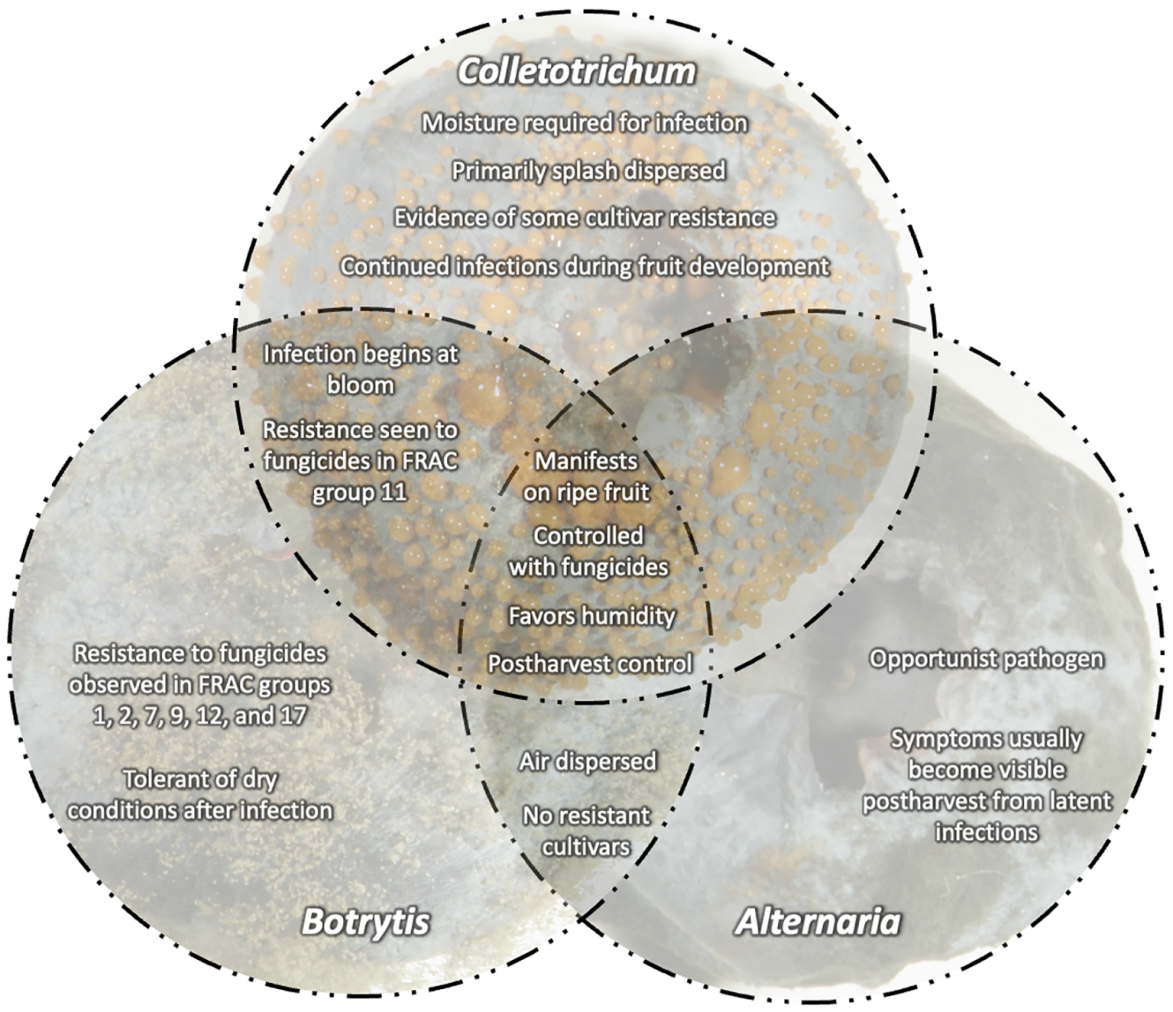
Central concepts for fruit rots caused by *Botrytis*, *Colletotrichum*, and *Alternaria* on blueberries. Overlapping disease characteristics including infection, dispersal, and fungicide resistance.

Since it is challenging to conduct controlled research at all locations for all environmental and cultural factors affecting blueberry growing conditions, the development of an online platform to share management practices is needed for blueberry fruit rots. By incorporating knowledge from different environments, we can foster shared learning and data collection with growers, extension agents, and researchers and build a comprehensive repository of best management practices in highbush blueberry. Incorporating considerations such as target organisms, fruit maturity, inherent fungicide efficacy, and the occurrence of fungicide resistance will allow for improved management of botrytis fruit rot, anthracnose fruit rot, and other fruit rots (**Figure 8**). Additionally, by incorporating economic factors such as the cost of management and environmental considerations, growers will be able to develop a comprehensive understanding of the implications of their chosen management strategy(ies). This multifaceted approach will ideally enhance the efficacy of disease management and foster sustainable practices by encouraging growers to weigh the environmental impact of their decisions. Furthermore, a shared environment offers the opportunity to discuss data on fungicide efficacy trials and will be a platform to provide decision support tools tailored to fruit rot management (e.g. degree day or phenology-based models). An online platform could empower growers to make informed choices, thereby mitigating the challenges associated with controlling blueberry fruit rots.

## Author contributions

KN: Writing – original draft, Writing – review & editing. CM: Writing – review & editing. MH: Writing – review & editing. JO: Writing – review & editing. JV: Writing – review & editing. YL: Writing – review & editing. KS: Writing – review & editing. VS: Writing – review & editing. PO: Writing – review & editing. TM: Writing – original draft, Writing – review & editing.
